# Multi-Criteria Optimization of Regulation in Metabolic Networks

**DOI:** 10.1371/journal.pone.0041122

**Published:** 2012-07-26

**Authors:** Clara Higuera, Alejandro F. Villaverde, Julio R. Banga, John Ross, Federico Morán

**Affiliations:** 1 Department of Biochemistry and Molecular Biology, Complutense University, Madrid, Spain; 2 Bio Process Engineering Group IIM-CSIC (Spanish National Research Council), Vigo, Spain; 3 Department of Chemistry, Stanford University, Stanford, California, United States of America; Centre for Genomic Regulation (CRG), Universitat Pompeu Fabra, Spain

## Abstract

Determining the regulation of metabolic networks at genome scale is a hard task. It has been hypothesized that biochemical pathways and metabolic networks might have undergone an evolutionary process of optimization with respect to several criteria over time. In this contribution, a multi-criteria approach has been used to optimize parameters for the allosteric regulation of enzymes in a model of a metabolic substrate-cycle. This has been carried out by calculating the Pareto set of optimal solutions according to two objectives: the proper direction of flux in a metabolic cycle and the energetic cost of applying the set of parameters. Different Pareto fronts have been calculated for eight different “environments” (specific time courses of end product concentrations). For each resulting front the so-called knee point is identified, which can be considered a preferred trade-off solution. Interestingly, the optimal control parameters corresponding to each of these points also lead to optimal behaviour in all the other environments. By calculating the average of the different parameter sets for the knee solutions more frequently found, a final and optimal consensus set of parameters can be obtained, which is an indication on the existence of a universal regulation mechanism for this system.The implications from such a universal regulatory switch are discussed in the framework of large metabolic networks.

## Introduction

For decades the regulation of metabolic networks at genome scale and its mechanisms has been studied to further our understanding of this process, especially after the massive increase of sequencing data during the post-genomic era. Cell regulation can be accomplished through two complementary strategies. Genetic regulation (genetic circuits) occurs at genome level, controlling the expression of certain genes. This regulation affects the presence or absence of enzymes in the metabolic network. On the other hand, post-transcriptional regulation operates in two forms: RNA mediated regulation and the dynamic control of enzyme activities. The latter is achieved by the activation or inhibition of certain enzymes by means of controlling metabolites, as is the case with allosteric regulation.

The idea that the metabolic pathways and regulation strategies that take place in a cell are the result of an evolutionary optimization process is widely accepted [1], [2]. Optimality principles have also been used to explain the structure of genetic networks [3], [4]. However, when it comes to defining the objective function that characterizes such evolutionary optimization, many uncertainties remain [5], [6], [7], [8]. Depending on the case in question, different criteria must be satisfied. Generally, in studies concerning metabolic networks the most frequently chosen objective is the maximization of metabolic reaction rates, or steady-state-fluxes. However, other criteria such as the maximization of the concentration of metabolites [9], [10], enzymes, or other metabolic performances could be considered. A more realistic alternative is to take more than one criterion into account, an approach that may be closer to the way in which nature has acted in the evolutionary process of optimization. In this way multi-criteria optimization plays an important role since it considers the simultaneous optimization of several objectives. Multi-objective optimization has already been used in different biological contexts. Handl et al published in 2007 an exhaustive review [11] about the application of multi-objective optimization in fields such as supervised and unsupervised classification of biological data, gene regulatory networks inference, sequence and structure alignment, protein structure prediction or optimization of biochemical processes among others. Several authors have performed preliminary research on the application of multi-objective optimization methods to reverse-engineering gene networks [12], [13], [14]. More specifically, this kind of optimization has also been used to search patterns or unique optimal solutions. In [15] the authors find that in different organisms the best-trade-off phenotypes were weighted averages of phenotypes specialized for single tasks. Furthermore Chubukov et al [16] found a pattern which relates the regulatory architecture of several yeast metabolic pathways to the gene expression response by searching a trade-off between two objectives: the cost of making a protein and the benefits of making it (its cellular function). This kind of works reveal that multi-objective optimization can, on the one hand contribute to find such patterns, and on the other hand to provide a closer approximation to natural evolutionary processes.

Unfortunately, finding a regulation design of a metabolic system as a result of an optimization process is an NP-hard problem in the majority of cases [7], [10]. The complexity and non-linearity of metabolic systems make the task of obtaining global optima in reasonable times impossible in many cases. In these situations the so-called stochastic global optimization methods, such as genetic algorithms or simulating annealing among others, can at least locate a near globally optimal solution, although they do not offer a full guarantee that the global optimum has been achieved [7].

In their work in 1995 [17], Gilman and Ross proposed a genetic algorithm (GA) to optimize the parameters governing a post-transcriptional regulation model. Their model studied the dynamic regulation of allosteric enzymes and idealized an animal cell that metabolizes blood glucose for energy as long as the glucose concentration in the blood is adequate, but synthesizes glucose for export if the glucose concentration in the blood drops too low. The end goal of Gilman and Ross was to find a regulation pattern which could perform optimally in different time-varying courses of concentrations of glucose inside and outside the cell. However, after running the GA on different courses no global winner was found. Their work showed the presence of “generalist solutions”, which performed well on one or several courses, and “specialist solutions”, which performed well on a single course but poorly on the others [17].

In this paper we take up again the challenge of finding a universal pattern of post-transcriptional dynamical regulation for this kind of model, set out by Gilman and Ross. We accomplish this goal through the study with different global optimization techniques and within the context of multi-criteria optimization. The latter has been carried out by calculating the Pareto-optimal [18] set of solutions according to two objectives. This set of solutions is considered to be a family of optimal solutions in the sense that it is not possible to improve one of the objectives without worsening the other; any choice of a unique solution would be a trade-off between both objectives.

The aim of this kind of optimization is to find a potentially universal mechanism of regulation of a specific metabolic network, by simulating the natural evolutionary optimization process.

## Materials and Methods

**Figure 1:**
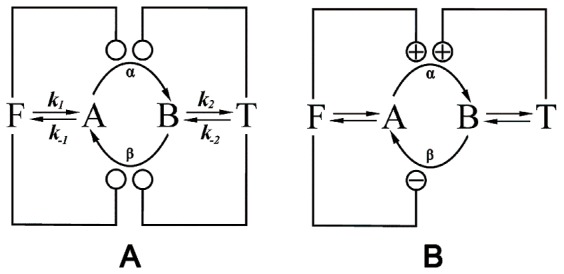
Diagram of the model. Substrate-cycle where enzymes *α* and *β* interconvert *A* into *B* (Fig. 1A), both regulated by external effectors *F* and *T*. Arrows indicate reactions, knobs indicate regulation. The kinetic parameters are: *k_1_* = 10^−2^ s^−1^; *k_-1_* = 8×10^−3^s^−1^; *k_2_* = 10^−2^ s^−1^; *k_-2_* = 4×10^−3^ s^−1^. For enzyme *α*, *V_ma_*_x_ = 1.6 mM s^−1^, *K_m_* = 1.5×10^−3^ mM. For enzyme *β*, *V_max_* = 3.5 mM s^−1^, *K_m_* = 2×10^−3^ mM. Fig. 1B shows an example of a regulation scheme of the model where symbol ‘+’ indicates activation and ‘−’ inhibition. In this case is activated by *F* and *T* because *R_α,F_* and *R_α,T_* are greater than 1 for the set of parameters taken as an example, and *β* is inhibited by effector *F* because *R_β,F_* is lower than 1. *T* has no effect on enzyme *β* because *R_β,T_* is 1.

In this work we have used the model examined by Gilman and Ross [17], depicted in Fig. 1A, which consists of a simple substrate-cycle where two metabolic intermediates (*A* and *B*) are interconverted by a pair of enzymes (*α* and *β*). These enzymes are regulated by two external “reservoirs” of metabolic species, and their concentrations are specified externally at any time (these variations of concentrations in a certain period of time are named “courses” from now on).

Since *α* catalyzes the conversion of *A* into *B* with rate *v_α_* and *β* catalyzes the conversion of *B* into *A* with rate *v_β_* the kinetic equations describing the temporal variation of these metabolic intermediates are described by the following differential equations:
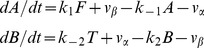
(1)


The enzyme-catalyzed reaction for *α* and *β*, in the presence of effectors, is of the form:
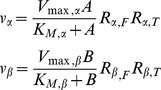
(2)where *K_M_* is the Michaelis-Menten constant and *V_max_* the maximum velocity of the corresponding enzyme. The factors modifying the intrinsic Michaelis-Menten rate expression are:



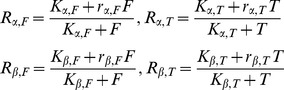
(3)The parameters *K_α,F_* and *K_α,T_* are the dissociation constants for the complex of enzyme *α*, and *r_α,F_* and *r_α,T_* are the ratios of the catalytic rate constants for the enzyme for the effectors *T* and *F* respectively. Similar notation is used for the enzyme *β*. Depending on whether the resulting expression of *R,* Eq. 3, is greater or less than 1 the corresponding enzyme, *α* or *β*, is activated or inhibited. A regulation diagram can be drawn from these statements. For example, if *R_α,F_* is greater than 1 the enzyme *α* will be activated by the effector *F* and the connection between *F* and *α* in the diagram will have a ‘+’ symbol. However, if *R_α,F_* is less than 1 the enzyme *α* will be inhibited by *F* and the connection between *F* and *α* in the diagram will have a ‘−’ symbol, while if *R_α,F_* is 1 the connection will not be shown since *F* has no effect on *α*. The same reasoning is applied to enzyme *β.* An example of a regulation diagram can be seen in Fig. 1B.

The regulation of the system, through the activation or inhibition of the enzymes *α* and *β*, is determined by the values of the set of these eight parameters (*K_α,F_, K_α,T_, K_β,F_, K_β,T_, r_α,F_, r_α,T,_ r_β,F,_ r_β,T_*). In order to optimize the flux response of the system the proper values of these parameters need to be selected. The main criterion for such optimization is the proper direction of the flux according to the system’s need. The response of the system should be able to provide an appropriate flux of both *F* and *T*, in response to a given external condition. For example, the system metabolizes blood glucose for energy as long as the concentration in blood is adequate but synthesizes glucose for export if the glucose concentration in blood is too low. In order to evaluate the system response Gilman and Ross, Eq. 4 in [17], formulated the following equation:
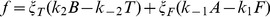
(4)Where the terms *(k_2_B−k_−2_T)* and *(k_−1_A−k_1_F)* represent the net fluxes into the reservoirs *T* and *F* respectively, and *ξ_F_* and *ξ_T_* represent their need state (expressions *ξ*_F_ and *ξ_T_* are described in figure 3 of [17]). If the concentration of *F* is below a specific target concentration, considered optimal, due to external variations, there will be a positive need state (*ξ_F_ = +*1), and the flux should flow from *B* to *A* in order to produce *F*. However, if the concentration of *F* is above the target concentration a negative need state will be induced (*ξ_F_ = *−1) and the flux should flow in the opposite direction (from *A* to *B*). The same applies to *ξ_T._*

**Figure 2:**
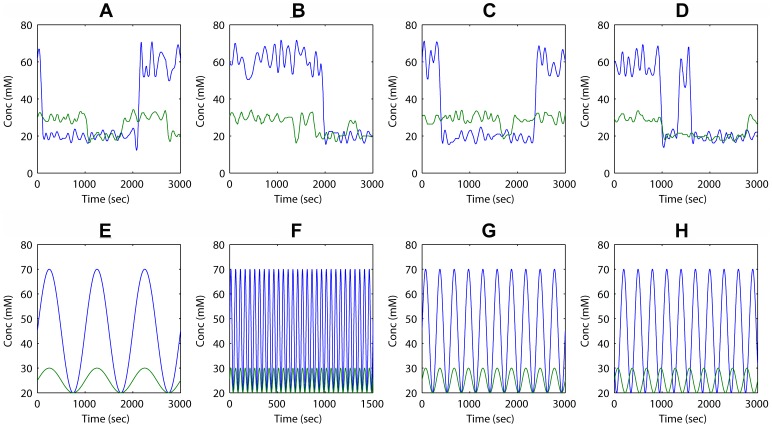
Time (sec) courses of external variations of concentrations (mM) of *F* (in blue) and *T* (in green). The first four courses *a–d* (A–D) are taken from figure 4 of [17] labeled as I, II, III and IV. The other four courses *e–h* (E–H) are obtained through the following sinusoidal equations: 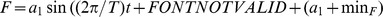 and 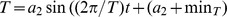, where *a_1_* and *a_2_* are the amplitudes, *t* is the time, *T* the period, *ϕ* the phase and *min_F_* and *min_T_* are the minimum values of *F* and *T*. The first two (*e* and *f*) differ in their period but have the same phase (*ϕ*  = 0); course *e* presents a high period (*T*  = 1000) while course *f* presents a lower one (*T* = 50). The two last sinusoidal courses (*g* and *h*) differ from each other in phase (for course *g*, *ϕ* = 0 and for course *h*, *ϕ*  = 10) and from the other two in period (*T* = 300). The concentrations of the reservoir species *F* and *T* vary within two regimes. For *F* centred at 60 mM and 30 mM and for *T* at 30 mM and 20 mM.

If the algebraic sign of both the net flux and the need state into a reservoir is the same, the flux will be directed in the proper direction, so in this equation a positive value of *f* is considered to be a good response.

In order to know how the network would behave in the different time-courses of *F* and *T* the integral of *f* over a period of time has been calculated. Eq. 5 gives some indication of the fraction of the period of time during which the flux was directed properly.
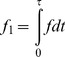
(5)


The energy “cost” for performing this operation during the period of time *τ* was calculated by Gilman and Ross [17] as a function of the operation of enzyme *α*, defined as:
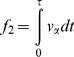
(6)


**Figure 3:**
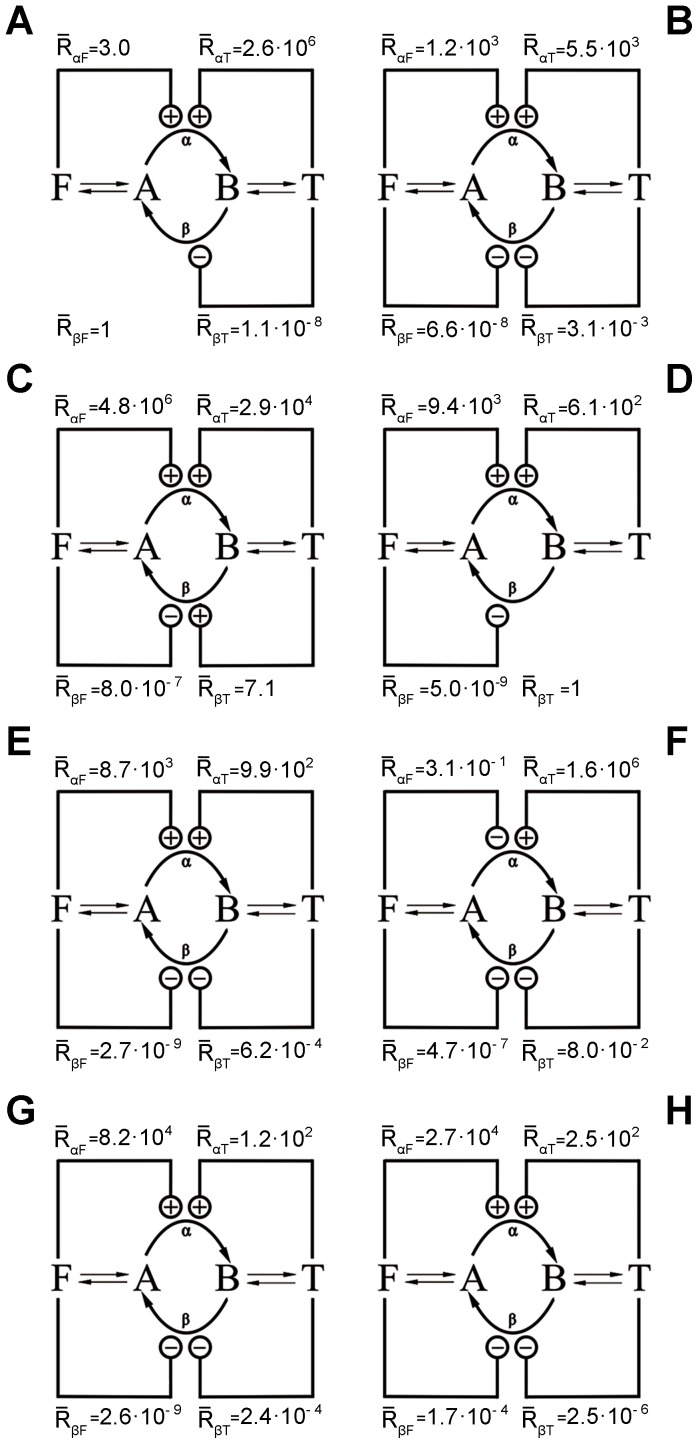
Regulation schemes obtained by the mono-objective methods for all the courses. Each diagram presents the values of 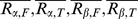 calculated with the resulting optimized parameters presented in Table 1.

**Table 1:** Resulting optimized parameters of the eight courses (*a, b, c, d, e, f, g* and *h*) after running the mono-objective optimization.

	K_α,F_	K_α,T_	K_β,F_	K_β,T_	r_α,F_	r_α,T_	r_β,F_	r_β,T_
**a**	9.4·10^5^	3.9·10^1^	1.3·10^4^	1.2·10^−10^	4.3·10^7^	4.2·10^9^	6.9·10^−7^	6.2·10^−9^
**b**	2.4·10^2^	4.5·10^4^	2.7·10^−9^	3.2·10^−10^	6.7·10^6^	9.99·10^9^	1.8·10^−10^	3.0·10^−3^
**c**	1.4·10^−8^	3.0·10^−6^	3.2·10^−8^	6.5·10^4^	4.8·10^6^	3.0·10^4^	7.2·10^−10^	1.6·10^7^
**d**	2.4·10^4^	1.4·10^5^	1.8·10^−10^	2.3·10^9^	5.1·10^9^	3.4·10^9^	4.4·10^−10^	4.4·10^7^
**e**	1.8·10^−2^	3.6·10^3^	1.1·10^−10^	1.5·10^−5^	1.3·10^4^	1.4·10^8^	2.0·10^−10^	3.9·10^−8^
**f**	3.2·10^−7^	5.4	5.3·10^−9^	5.4·10^−10^	3.2·10^−1^	3.6·10^8^	3.4·10^−7^	8.0·10^−2^
**g**	9.3·10^−3^	2.0·10^6^	1.0·10^−10^	1.0·10^−10^	1.0·10^5^	1.0·10^10^	1.0·10^−10^	2.5·10^−4^
**H**	4.9·10^2^	1.7·10^5^	3.5·10^−8^	4.3·10^−6^	3.4·10^8^	1.3·10^9^	1.0·10^−7^	5.2·10^−6^

### Mono-objective Global Optimization

The nonlinearity and frequent multimodality of this kind of model make the optimization of its parameters a difficult task for traditional optimization methods, which are very sensitive to the initial values. Such problem models can contain several local optima, hence if the initial values are far from the global optimum it is difficult to assure a convergence towards it [8]. A robust alternative for solving complex-process optimization problems is to use global optimization methods [7], [19]. These kinds of methods can be roughly divided into two classes: deterministic and stochastic. Deterministic methods guarantee finding the global optimum under certain conditions. Their drawback is that the computational effort they require increases very fast with the problem size [20]. On the other hand, stochastic methods are based on probabilistic algorithms and do not offer the guarantee of finding the global optimum; however, it has been proved that they provide excellent results in solving complex-process optimization problems [19], [20] in reasonable computation time.

In [17] a GA which belongs to the class of global stochastic optimization methods was used. The authors combined the flux response, Eq. 5, and a weighted cost, Eq. 6, by means of a single objective function:
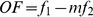
(7)


A high value of *OF* is obtained not only when the network responds properly to changes of external concentrations but also when it does so at a low biological cost. Therefore a set of parameters must be found that maximizes *f_1_* and minimizes *f_2_*, resulting in an optimal solution which would be a trade-off between a proper performance of the network (*f_1_*) and the cost (*f_2_*), merging these two concepts into one equation. As asserted in [17], the GA procedure did not always find the global optimum, indeed for each run of the method a different value of *OF* was found making it difficult to assure the convergence towards an optimum. In this paper we have performed a Mono-objective study of the system using three different stochastic global optimization methods: a variation of the GA used by Gilman; the enhanced scatter search SSm method described in [19]; and the multistart clustering method GLOBALm [20]. The scatter search method uses a relatively small population size, partially chosen by a quality criterion from an initial set of diverse solutions. It also performs systematic combinations among the population members. It is interesting to note the similarities and differences between scatter search and the original genetic algorithm (GA) framework. Both can be regarded as “population based” or “evolutionary” approaches, since both incorporate the idea that a key aspect of producing new elements is to generate some form of combination of existing elements. However, GA approaches are based on the idea of choosing parents randomly to produce offspring, and on using randomization to determine which components of the parents should be combined. In contrast, the scatter search approach does not place so much emphasis on randomization. Instead, the approach is designed to incorporate strategic responses, both deterministic and probabilistic, that take account of evaluations and history of the search. These components result into a more efficient search than GAs. On the other hand, GLOBALm is an extension of the multistart clustering algorithm for global optimization, incorporating new key features, including an efficient mechanism for handling constraints and a robust derivative-free local solver. The multistart clustering framework is based on starting with the generation of a uniform sample in the search space (the region containing the global minimum, defined by lower and upper bounds). After transforming the sample (e.g., by selecting a user set percentage of the sample points with the best function values), the clustering procedure is applied. The aim of the clustering step is to identify points from which the local solver will lead to already found local minima. Then, further local searches are started from those points which have not been assigned to a cluster, and the process is repeated until a stopping criterion is satisfied. The three methods have been applied to eight different time courses of external variations of concentrations of *F* and *T*, which are pictured in Fig. 2. The first four courses *a-d* (Fig. 2A, B, C, D) have been taken from [17] and the other four courses *e-h* (Fig. 2E, F, G, H) are sinusoidal periodical variations of *F* and *T* with different frequency, amplitude and phase.

**Figure 4:**
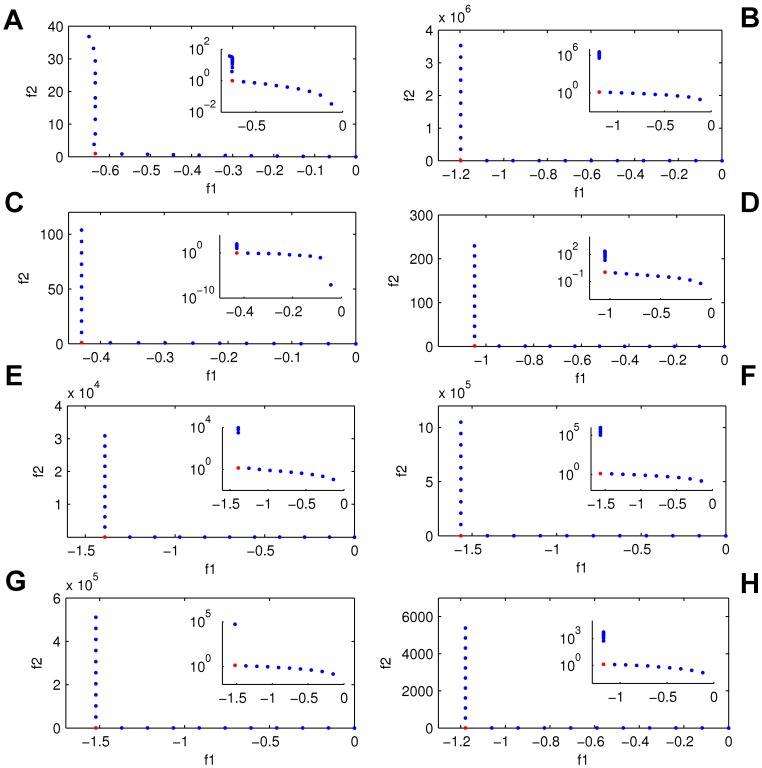
Pareto fronts obtained for the two objective functions. Flux response (*f_1_*) vs. cost (*f_2_*), for the eight courses. The insets are semi log plots. Each point corresponds to a set of the eight parameters. Red points indicate the knee point of each front.

### Multi-objective Global Optimization

The performance of the network in terms of flux response and energy cost can be analysed independently, so that a more desirable and realistic approach would be to consider the simultaneous optimization of these two criteria. In this case the result would not be a unique solution, but a set of solutions representing the trade-off between both objectives [21]. This approach is called multi-objective (or multi-criteria) optimization (MO), and despite being better able to cope with complex models, few applications are found in the systems biology literature in comparison with other scientific and engineering fields [22].

The simultaneous optimization of multiple objectives differs from traditional mono-objective optimization in that if the objectives are in conflict with each other, the solution to the optimization problem will not be unique; instead, there will be a family of solutions known as a Pareto-optimal set [18]. For the case in which there are two objectives, *f_1_* and *f_2_*, the Pareto optimal set is a set of solutions in which no improvement can be obtained for *f_1_* without making *f_2_* worse, and vice versa. In this sense, no point from this set can be said to be better than another; hence, in the absence of any further information about the problem, all Pareto-optimal solutions (which may be an infinite number for continuous problems) are mathematically equivalent. If one is interested in achieving only one final solution, there is a need for a decision-making process that allows one of the solutions in the set to be selected, using additional information. The choice of a particular solution is often subjective or difficult to express in mathematical terms, and it is therefore difficult to obtain systematically. However, the Pareto front of some multi-objective optimization problems shows a solution that can be considered to be the best compromise, i.e. the optimum of the front. These solutions are called “knee” points [23]. They are characterized by the fact that even a small improvement in one of the objectives (say *f1*) would come at the cost of a much worse value of the other objectives (in this case *f2*).

One of the advantages of the Pareto front perspective is that it allows the representation of the solutions within a diagram. Since the present model has two objectives, the solutions can be displayed in a 2D diagram dividing the graph in different regions. For instance, a set of solutions which control the flux properly but at a high cost will be situated together on one side of the diagram while solutions which do not control the flux so well but minimize the cost optimally will be placed on the opposite side, leaving the solutions which represent a trade-off between the two objectives in the middle. Since the solutions are laid out in regions along the Pareto front, with this approach we were able to organize them in a graphical and more visual way, thus obtaining a wider perspective in the study of optimization applied to biochemical systems.

Optimizations were carried out with the NBIWT weighted Tchebycheff method presented in [9]. NBIWT is a multicriteria optimization method that ensures an even spread of solutions in the Pareto front without the need of user-specified weights. It is based on the normal bounday intersection (NBI) method [24] with extensions based on the weighted Tchebycheff method [25]. NBIWT also incorporates several stochastic local and global optimization solvers so it is able to handle both convex and non-convex Pareto fronts. Overall, it provides the user with a robust and efficient method of computing Pareto fronts without the trying of weights or other tuning parameters for the different objective functions.

An optimal (Pareto) set was computed for each of the eight courses for the cost, Eq. 6, and flux response, Eq. 5, simultaneously. The NSGA-II [26], [27] method was also used initially but resulted in worse results than NBIWT.

**Figure 5:**
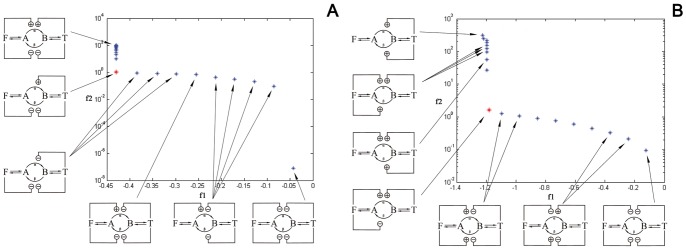
Semi log plots of the Pareto fronts for courses *c* (A) and *d* (B). Each point in the front corresponds to an optimal solution for *f_1_* and *f_2_* given by the estimated set of the eight control parameters. The small diagrams represent the corresponding regulation schemes deduced from each set of parameters. The red point corresponds to the knee point.

**Figure 6:**
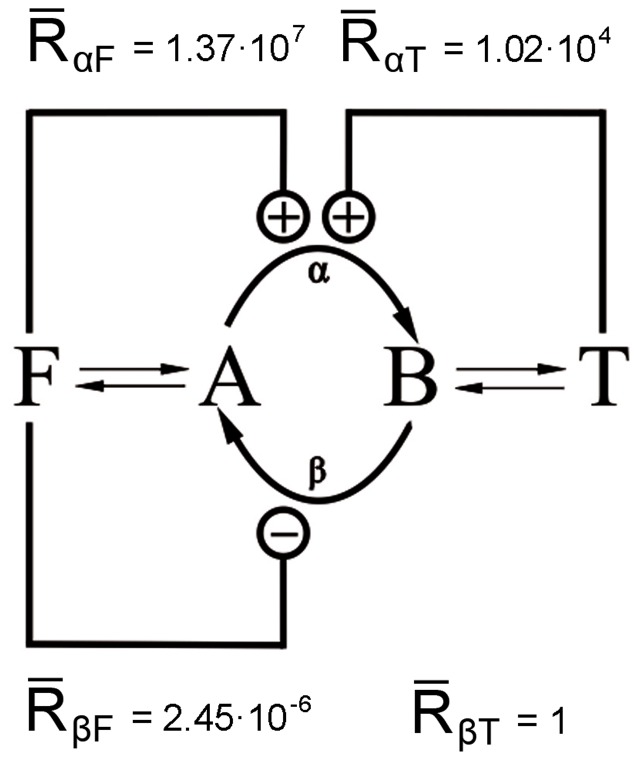
Resulting regulation scheme drawn from the consensus set of parameters. Enzyme *α* is activated by both effectors *F* and *T* (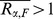 and 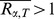 ) whereas enzyme *β i*s inhibited (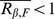) by *F* and *T* has no effect on it (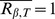). The corresponding optimized parameters are: *K_α,F_* = 1.5·10^−2^, *K_α,T_* = 5.5·10^3^, *K_β,F_* = 9.2·10^−8^, *K_β,T_ = *3.7·10^7^, *r_α,F_ = *1.9·10^7^, *r_α,T_* = 2.3·10^9^_,_ *r_β,F_ = *1.6·10^−7^_,_ *r_β,T_* = 3.65.

## Results

### Mono-objective Global Optimization

Following [17], a value of m = 10^−3^ has been used for the objective function *OF,* Eq. 7. Ten optimization runs were repeated using the three methods on each of the three courses. To allow a fair comparison between the three methods, we ran them with equivalent setting parameters: in the case of GA a population of 100 individuals and 100 generations was used which represents a total of 10,000 evaluations. The same number of evaluations was set for SSm and Globalm. The optimum was achieved in less than 1000 evaluations, a relatively small number. The remarkable result was that the same *OF* optimal value was obtained by each method for each course. No significant differences were found between the three of them in terms of computation time or convergence towards the optimum. As stated above, stochastic global optimization methods do not generally guarantee convergence to the global optimum. However, the fact that the three different methods reached the same objective function value in several runs strongly suggests that in this case the global optimum has been achieved.

In terms of regulation, due to the nature of the system, certain degeneracy in the solutions could be expected since different schemes of regulation could achieve the global optimum. However it is worth mentioning that after running any of the algorithms ten times in a particular course the regulation scheme corresponding to the optimal solution obtained was in most of the cases the same. Nevertheless, among the different courses the resulting regulation scheme can be different, as shown in Fig. 3. In order to obtain these schemes, since *R,* Eq. 3, depends on the values of *F* and *T* which vary during time, we compute their averaged values 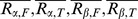 for maximum and minimum concentrations of *F* and *T*, (60 mM and 30 mM, and 30 mM and 20 mM respectively) these values are also shown in Fig. 3. The optimized parameters of the different courses are shown in Table 1.

**Figure 7:**
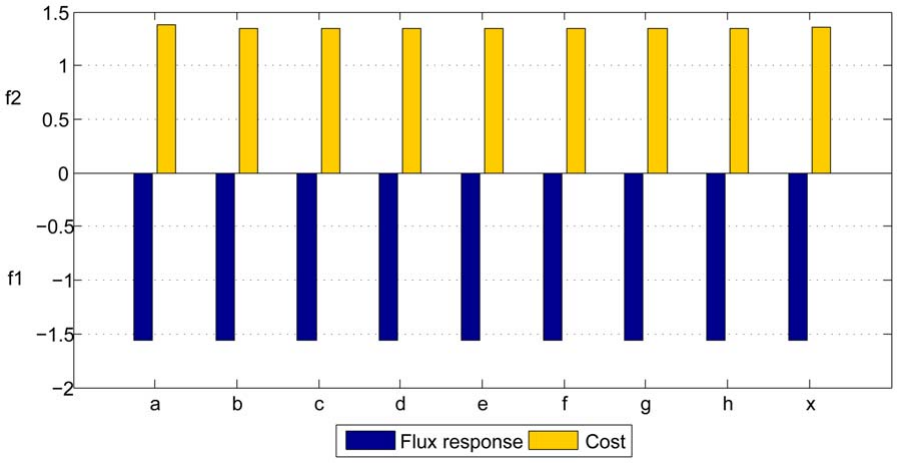
Evaluation with knee parameters and consensus parameters. Values of flux response (*f_1_*) and cost (*f_2_*) for course *f* evaluated with the sets of parameters corresponding to the knees obtained for the different courses and with the consensus set of parameters, represented as x.

**Figure 8:**
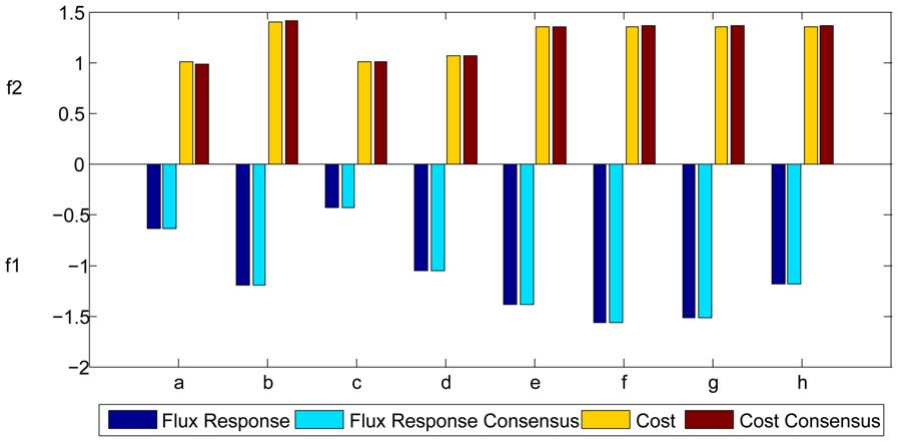
Cross-course comparison. Flux response (*f_1_*) and cost (*f_2_*) for the different courses evaluated with their optimal parameters (in dark blue and yellow) and with the consensus set of parameters (in light blue and brown).

### Multi-objective Global Optimization

After applying the NBIWT weighted Tchebycheff method on each of the eight different time courses we obtained eight fronts of solutions. Strikingly, each of them exhibited the same kind of Pareto front characterised by containing a clear knee point, which represents the ideal trade-off between the two objectives: in this case that solution combines a high flux response (*f1*) at a low cost (*f2*). Fig. 4 shows the eight Pareto fronts and the corresponding knee point.

Each of the solutions (points) of the Pareto fronts corresponds to a set of parameters (four *K’s* and four *r’s*). The regulation diagrams corresponding to the different solutions of the front are shown in the small diagrams of Fig. 5 for courses *c* (Fig. 5A) and *d* (Fig. 5B). A certain similarity in terms of regulation schemes between the knee points of the different courses can be expected, since they are optimal solutions. In this way it would be possible to find a scheme (i.e., a set of parameters) which would be optimum for every course, however, although it can be noticed that the regulatory schemes of the knee points maintain some basic similarities, they are not identical.

There are some remarkable similarities among certain regions of the Pareto fronts within different courses that can be observed in Fig. 5A and 5B, for instance in the right-hand side of the fronts many of the solutions presented a scheme where both enzymes *α* and *β* were inhibited. This would explain the fact that these solutions have a very low value of *f_2_*, since *f_2_* (Eq. 6) is directly related with *v_α_*, and if this enzyme is inhibited the value of *f_2_* will be low. In contrast to this, on the top left-hand side the solutions of the fronts presented a high value of *f_2_* and the most frequently found scheme was the one in which *α* was activated by the two effectors.

Interestingly, it was found that if the different knee points were interchanged within the different time courses the resulting behavior was also optimal in each of them. For example, the parameters set corresponding to the knee point of course *a* also yielded optimal values of *f_1_* and *f_2_* for the other seven courses, and that happened with every knee point. This result suggests the existence of an underlying universal regulation pattern. In order to find such pattern, several runs of the method NBIWT were carried out for each course and the regulation scheme of the knee point of each run was studied. It was observed that between different runs of a course the regulation scheme corresponding to the knee was slightly different, however it was noticeable that certain regulation pattern was more frequent than others, we consider this a consensus regulation scheme for this system, see Fig. 6. The consensus set of parameters was calculated averaging the parameters belonging to the knee points which presented this scheme. To this end we calculated the average of each of the eight control parameters individually (*K_α,F_, K_α,T_, K_β,F_, K_β,T_, r_α,F_, r_α,T_, r_β,F_, r_β,T_,*).
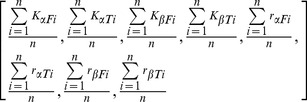
(7)where n is the number of times that the consensus scheme has been observed. The resulting consensus set of parameters resulted to be as good as the optimal as well proving that a universal set of parameters can be achieved.

Fig. 7 represents the value of flux response (*f_1_*) and cost (*f_2_*) for course *f*, evaluated with the different knees of all the courses (optimal solution obtained for each course) and also with the consensus set of parameters. Similar results were obtained for the other seven courses. Remarkably, these values are very similar to each other, indeed the deviation of the different values of flux response for a single course evaluated with its optimal set of parameters, the other knee solutions, and the consensus set, is always less than 0.006 (that corresponds to a maximum deviation of 0.7%). In the case of the cost the deviation is always less than 0.019 (maximum deviation of 1.38%). Fig. 8 shows a comparison of the flux response and cost obtained for each course run with its optimal set of parameters and the values obtained with the consensus set of parameters. It is noteworthy that there are practically no differences.

The regulation scheme corresponding to the consensus set of parameters is depicted in Fig. 6. Enzyme *α* is activated by both effectors *F* and *T*, favoring the production of *B*, and enzyme *β* is inhibited by *F*. These schemes, where the enzymes are regulated by products and substrates of the reaction, are frequent in metabolism [28], [29]. Specifically, the universal pattern obtained in this paper corresponds to several examples of substrate-cycles found in literature. One relevant example is the conversion of fructose 6-phosphate (F6p) into fructose 2,6-bisphosphate (F2,6BP) described in textbooks, e.g. [28]. In this cycle of transformation (F6P 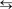 F2,6BP) the kinase activity of the phosphofructokinase (PFK2) is activated by its substrate (F6P) and the activity of the phosphatase is inhibited by the product (F6P). Finally, the PFK2 is activated by the product F2,6BP. A similar behaviour is also observed in the regulation of gluconeogenesis and glycolysis in the liver (figure 16.28 of [28]), where there is an activation by substrate of the PFK mediated by AMP and F2,6BP and also an inhibition of FBPase by the same metabolites. This result reinforces the natural appearance of reciprocal feedback seen in multiple instances of biochemical networks.

## Discussion

The starting point of this work was the hypothesis that the regulation mechanisms of metabolic networks are the result of an evolutionary process of optimization. The idea that nature carries out optimizations in terms of metabolic regulation led us to search for existing universal regulatory patterns. In this paper we have investigated the existence of a global optimal solution in a substrate-cycle previously presented in [17] which was optimized using a GA. Since GAs are stochastic global optimization methods, they do not provide guarantees of convergence to the true global solution. When Gilman and Ross [17] performed optimization runs on different environments (i.e., time courses of end point species concentrations), they were surprised that they did not find a global winner. Instead, solutions found to be optimal for one of the courses were not optimal for the other courses: they were “specialists” but not “generalists”.

A first objective of the research reported here was to investigate this aspect further by, on the one hand, reproducing the original results of [17] using a modification of the GA and, additionally, two state of the art global optimization methods: SSm [19] and GLOBALm [20]. All these methods reached essentially the same solutions, strongly suggesting that the results presented here are very likely global optima.

As a second objective, we wanted to find a unique regulatory scheme by means of multi-criteria optimization. Here we have been able to find a generalist solution by switching from mono- to multi-criteria optimization. Instead of optimizing with respect to an objective function consisting of a fixed combination of the performance and cost terms (*f_1_* and *f_2_*) we applied a multi-objective strategy (NBI-based weighted Tchebycheff, NBIWT) as presented in [9]. Essentially, this technique generates an even spread of points on the Pareto front, which correspond to the different relative weights of the two objective functions. As a result, instead of a single solution we found, for each environment, the set of Pareto-optimal solutions (set of optimal compromises for the two costs considered). Then, realizing that the Pareto fronts of all the environments exhibited a clearly defined knee point, we identified those solutions as the ones providing the best trade-offs between the two objectives. It should be noted that these solutions cannot be found systematically using a classical mono-objective optimization scheme.

Interestingly, we found that although these solutions corresponded to different regulation schemes, they performed optimally not only in the environment for which they were optimized, but also in the other environments. Repeating several times the multi-criteria optimization for each course we found a frequent optimal pattern of regulation, a regulation scheme that balances performance and cost optimally in every environment for the system considered. This can be seen as an indication on the existence of a universal regulation mechanism for substrate-cycles which are very frequent in metabolism. Several examples can be observed in the literature [28], [29]; of special relevance is the PFK2-FBPase2 cycle [28], which has exactly the same regulation pattern that we have obtained by means of multi-criteria optimization. It is worth mentioning that resulting optimal trade-off solution (knee point) presented multiple global solutions (different regulation schemes with the same trade-off in the space of cost functions). This multiplicity is typical of multicriteria problems where the cost functions are of the integral type.

This approach can be easily scalable to larger networks composed of more than one regulatory unit, such scalability poses no major problems other than increased computational requirements. The scatter search method scales quite well with problem size and has been successfully used in optimizations of several hundreds of decision variables. The increased computational cost can be handled by exploiting parallelization strategies. Versions of the scatter search and NBIWT solvers exploiting high performance computing hardware are being developed and therefore will enable the application to larger networks which could allow a more systemic optimization of metabolic systems. It should be noted as well that the approach presented is general in the sense that can be applied to other contexts (such as e.g. different individual cost functions, additional constraints, etc.) and can therefore be tailored to arbitrary multi-criteria optimization problems. Besides, the implications of the work presented in this paper go beyond the analysis of regulation based on optimality principles. For example, we can use a similar multi-criteria optimization scheme for the optimal design of biological circuits, as considered in synthetic biology. Optimization methods have recently been used for such designs, as discussed in e.g. the review by Marchisio and Stelling [30]. We suggest increasing the robustness and feasibility of these designs by adopting a multi-criteria framework similar to the one presented here.
