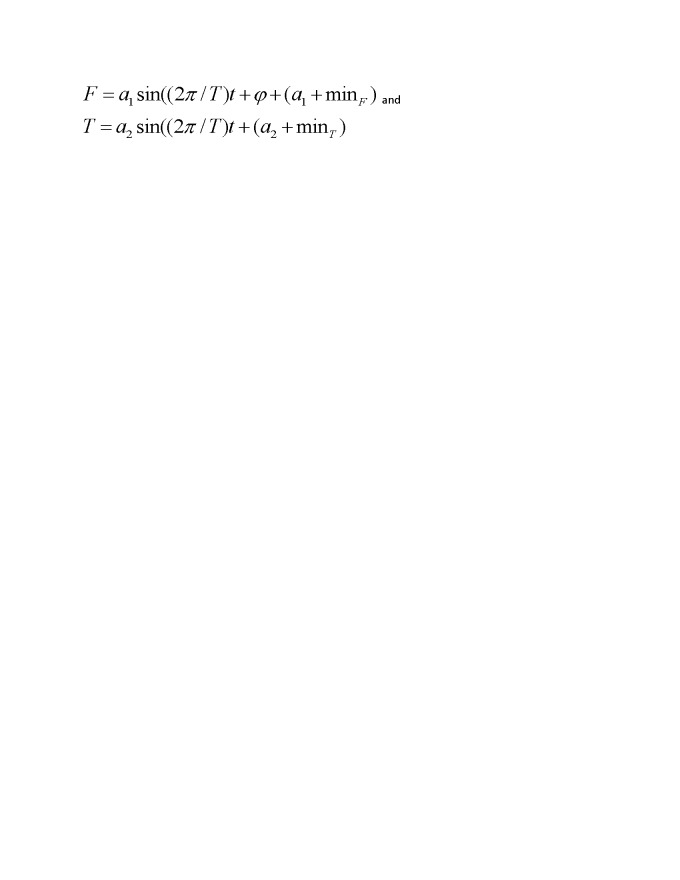# Correction: Multi-Criteria Optimization of Regulation in Metabolic Networks

**DOI:** 10.1371/annotation/ab5c1e64-2c46-421e-a1f7-e79cc415534d

**Published:** 2012-11-14

**Authors:** Clara Higuera, Alejandro F. Villaverde, Julio R. Banga, John Ross, Federico Morán

There were equation formatting errors in the Figure 2 legend. The correct equations can be viewed here: 

**Figure:**